# Supplementary material for: Viral disease outcomes are indistinguishable between experimentally infected bats and rodents
Source: PLoS Biol. 2026 Oct 29;24(10):e3004053. doi: 10.1371/journal.pbio.3004053 (PMC13743028; doi:10.1371/journal.pbio.3004053)
Supplement: Supplementary file 3 [file pbio.3004053.s003.pdf]

PLOS Biology – Response to Nature Microbiology editor and reviewer comments

28th May 2026

Dear Dr Farrell,

Thank you for your patience while your manuscript "Viral disease outcomes are indistinguishable between experimentally infected bats and rodents" was under peer review at Nature Microbiology. It has now been seen by our referees, whose expertise and comments you will find at the end of this email. In the light of their advice, we have decided that we cannot offer to publish your manuscript in Nature Microbiology.

From the reports, you will see that while they find your work of some potential interest, the referees raise concerns about the advance your findings represent over earlier work and the strength of the novel conclusions that can be drawn at this stage. In particular, the reviewers have issues with the analyses and say that the datasets are insufficient to address the questions in place. Unfortunately, these criticisms are sufficiently important as to preclude publication of your work in Nature Microbiology.

Although we cannot offer to publish your manuscript, I have discussed your manuscript and the reviewers' comments with our colleagues at Nature Communications and Communications Biology. I am glad to say that both journals would be happy to offer a path to publication.

Nature Communications would send the appropriately revised version out for further review if you transfer the revised manuscript. In particular, they ask you to clarify reviewer #3's concerns about the potential bias with viral loads and inoculation routes. Further, they ask you to temper too strong conclusions along the lines listed by all referees, to clearly outline the limitations of the analysis and derived conclusions, as well as provide an outlook about what type of experimental data would be necessary to resolve analytical issues in the future. Should you wish to have your revised paper considered by Nature Communications, please use the link to the Springer Nature manuscript transfer service below the revision is ready, and include a point-by-point response to the reviewers' concerns. Your handling editor at Nature Communications would be Dr. Madlen Luckner (madlen.luckner@nature.com). If there is anything you would like to discuss before transferring the paper and its reviews, please don't hesitate to contact her by e-mail. Please note that Nature Communications is a fully open access journal. For information about article processing charges, open access funding, and advice and support from Springer Nature, please consult the Nature Communications Open Access page (www.nature.com/ncomms/open_access/index.html).

I have also consulted with Dr. George Inglis at Communications Biology, a selective open access journal from Nature Portfolio. **They are very interested in your study and would**

be delighted to send a suitably revised manuscript back to the original referees, if available, should you transfer it to them. Specifically, they ask that you qualify your conclusions in light of the comments from Reviewer #3, and address all remaining discussion points from the reviewers. To have your revision sent back to review by the editors at Communications Biology, please use the link to the manuscript transfer service provided below. If you would like to discuss your manuscript with them directly, you can contact them at (george.inglis@us.nature.com). If you decide to submit your revised manuscript to Communications Biology, please ensure that your revision is accompanied by a detailed point-by-point response to all the reviewer comments and a cover letter summarizing the changes made to the manuscript. Please also include completed Life Sciences Reporting Summary and modify your manuscript if needed, to comply with the instructions in these documents. Communications Biology is a fully open-access journal and an article processing charge will apply to any papers accepted for publication. For further details about Communications Biology, including their editorial policies and information about open access funding, please visit the journal website at https://www.nature.com/commsbio/ .

To transfer your manuscript please use our manuscript transfer portal. You will not have to re-supply manuscript metadata and files, unless you wish to make modifications. For more information, please see our manuscript transfer FAQ page.

I am sorry that we cannot be more positive on this occasion, but hope that you find the referees' comments helpful when preparing your paper for resubmission elsewhere.

Yours sincerely,
Paula Jauregui

Paula Jauregui, PhD
Senior Editor
Nature Microbiology

****
Reviewer Expertise:

Referee #1: Innate immunity against virus, bats
Referee #2: Bats immune response
Referee #3: Ecology and evolution, bats, math models

Reviewers Comments:

Reviewer #1 (Remarks to the Author):

Once again, I enjoyed reading this manuscript and feel it is an important question that should be explored. The authors have clearly worked hard to address prior reviewer feedback, and the revised manuscript is substantially improved. The reframed title and abstract, expanded analyses, and added clarifications around experimental design and coevolutionary context have strengthened transparency and helped align the claims with the available data. However, the manuscript's framing and conclusions still extend beyond what the compiled data can robustly support.

1. I appreciate the authors have clearly defined tolerance and resistance and justify the use of disease outcomes as a measure of tolerance. Tolerance is generally defined as reduced host damage at a given pathogen burden. However, the analyses rely entirely on author reported clinical signs, pathology, and mortality, without quantitative measures of viral burden or host fitness costs. Without pathogen load or damage per load relationships, conclusions about tolerance mechanisms are necessarily indirect.
I would suggest reframing the manuscript more explicitly around reported disease outcomes following experimental infection, rather than tolerance itself.

We agree that quantifying tolerance from the existing literature on experimental infections is problematic and have followed the reviewer's suggestion to re-frame the manuscript on disease outcomes, which the current literature on bat immunology has suggested is relevant for putatively unusual patterns of resistance and/or tolerance in bats.

2. The authors appropriately acknowledge that the experimental infection literature is heterogeneous with respect to viruses, doses, routes of inoculation, passage history, and reporting standards. While the hierarchical modeling framework accounts for many of these factors, the absence of paired comparisons (same virus, similar dose, similar route across host taxa) still limits causal interpretation.
I would suggest emphasizing limits on inference arising from unavoidable study heterogeneity and acknowledging that the results address the weight of evidence in the existing literature.

We have now added comments in the Discussion and Implications for future research to further emphasize our results are based on currently available data, which included large variation in study design.

3. The manuscript concludes that the findings do not support the idea that bats will be an unusually rich source of future biomedical breakthroughs. This statement risks overextension: organism level disease outcomes in experimental infections do not directly bear on the translational value of specific molecular immune mechanisms, some of which have already demonstrated utility in non bat systems.
I would recommend avoiding general statements about the relative value of bats for biomedical discovery. A more balanced framing acknowledging that mechanistic insights

may be valuable even in the absence of generalized tolerance would strengthen the manuscript.

We agree with the reviewer that organismal level disease outcomes may or may not signal the future therapeutic value of any single molecular mechanism exploited. Our manuscript is questioning what the field more broadly has suggested, that – all else constant – the putatively unusual ability of bats to co-exist with viruses could signal the presence of an unusually potent or broad suite of antiviral mechanisms which might be exploited therapeutically. In other words, we are testing the current evidence for atypical host-virus interactions, not the claim that unusual interactions should be expected to have higher biomedical value.

In the revised abstract, we now explicitly state the value of mechanistic insights into bat immunity and clarify that our manuscript challenges the "*premise underlying the idea"* that bats will be an unusually rich source of future biomedical breakthroughs (Lines 19-20).

We have also expanded our Implications for Future Research section to offer a more balanced framing about the potential value of bat immune systems for antiviral discovery (Lines 319-325):

"The belief that bats evolved exceptionally generalized or unusually potent mechanisms to suppress virus-induced disease largely underpins the aspiration that bats will be an unusually rich source of broad-acting or potent antivirals. The incompatibility of the currently available data with this premise questions whether the translational value of bat immunity should be expected to be disproportionately high relative to other host groups. Nevertheless, our results do not diminish the potential biomedical value of uncovering the mechanisms by which animals evade severe disease, and useful antivirals may be developed from bat immune systems in the future."

Reviewer #1 (Remarks on code availability):

This is not my area of expertise

Reviewer #2 (Remarks to the Author):

The authors have satisfactorily addressed this reviewer's criticisms.

Reviewer #3 (Remarks to the Author):

Thanks to the authors for their careful consideration of my comments from last year. I find

the new manuscript more balanced and thoughtful. However, I still believe that the shortcomings of this dataset are not resolvable to address the questions that have been asked. The main issues with the paper can be summarized as follows:

1. Viral tolerance is still not (and likely cannot be) addressed with the data in hand: While the authors have removed the term 'tolerance' from the title and abstract, it remains the focus of the entire paper and is heavily emphasized in the introduction (including the stated goals of the research) and the discussion. The authors state that in the absence of actual measurements of viral tolerance (which require quantification of both variation in fitness costs across variation in viral load), they 'operationally define' it based on the extent of author-reported disease from viral infection. While I am sympathetic to the limitations of the available literature, this should inspire empirical work to collect these data rather than potentially erroneous attempts to draw tolerance conclusions from inadequate evidence.

As mentioned above in response to Reviewer 1, we have removed our operational definition of tolerance and instead focus the manuscript on disease outcomes as an output that the field has consistently argued is a relevant reason to think that tolerance/resistance is distinctive in bats.

2. Unrealistic viral loads in experimental infection: As stated previously, the vast majority of the experimental infections analyzed in both rodent and bat taxa use viral loads that are too high to be relevant to natural infection. While the authors claim that the lack of a host order effect in models controlling for pathology by dose is still noteworthy, this entirely depends on the shape of the host fitness-viral dose curve, for which the shape is often unknown and frequently nonlinear. If all doses considered in this analysis cluster at the high end of the host fitness-viral load relationship, the authors might miss important regions of the curve that are fundamental to understanding tolerance. Theoretical work has long recognized that pathogen tolerance might function in a threshold sense, whereby hosts might tolerate infections up to a threshold pathogen load, after which virulence scales proportionally with pathogen load in both tolerant and nontolerant hosts (e.g. Miller, White, Boots. Evolution 2006). Recent theoretical work has also suggested that intermediate inoculum doses may lead to prolonged infections which could be important in understanding zoonotic spillover (Graham and Regoes 2024). As stated previously, understanding tolerance requires quantification of host fitness upon exposure to the same pathogen across multiple different pathogen loads, and this study does not address this.

As above, we have re-framed our manuscript around disease outcomes rather than a formal or operational measurements of tolerance. In the Introduction we have added clarity around this point (Lines 61-69):

"We note that despite the informal use of the term "tolerance" in the bat virus literature when referring to the outcomes of experimental and natural viral infections, formally measuring tolerance would require non-invasive quantification of viral loads in

physiologically relevant tissues and prohibitively expensive long-term monitoring of fitness costs in infected hosts. It is therefore unclear that tolerance has or can be realistically measured from experimental or natural infections (Medzhitov et al. 2012). We therefore frame our analysis around the occurrence and severity of outcomes following viral infection based on the extent of author-reported disease (including clinical and sub-clinical pathologies). Because we are interested in disease outcomes in compatible host-virus pairs, we restrict our analysis to inoculations in which the original authors deemed hosts to be susceptible".

Our manuscript presents an analysis of currently available data on experimental inoculations, and we suggest that future studies investigating natural immune responses aim to use realistic doses and inoculation routes, when known (Lines 342-348):

"When known, studies focused on comparing natural immune responses should also aim to use realistic doses and inoculation routes. Our finding that inoculation dose and study size influence observed disease outcomes also suggests that some studies showing limited disease may be artifacts of insufficient powering of experiments for disease detection. This emphasizes the need for larger experiments to detect disease outcomes, and the value of synthesizing data across multiple experiments to detect biases which would be missed by single studies and to make generalizable inferences despite them, as we have done here."

3. Lack of sufficient analysis of potential biases by inoculation route: The authors attempted to address my prior criticism of the lack of consideration of the importance of viral inoculation route (in addition to dose) in driving pathology outcomes by including inoculation route as a co-variate in some individual-level models and by rerunning analyses removing inoculations into the brain which were disproportionately conducted in bats. I was firstly confused by multiple supplemental plots that do not show inoculation route as a predictor variable in corresponding models (e.g. Fig S21-23 and S26-32) – were these analyses undertaken without considering inoculation route? I can't see any reason why this should ever be left out of any of the paper's conclusions if it is possible to include in the analysis (e.g. if the level of analysis is individual). In addition, for all analyses, including those at the species level, these should be undertaken by comparing only cross-species pathology within a given inoculation route while controlling for viral load. Qualitative review of Fig S8 suggests that oronasal routes of infection (which seem the closest inoculation route to a natural infection) might produce less severe pathology in bats than rodents but this will need proper consideration with statistical rigor, of course accounting for viral load. Similarly, natural infection routes appear to result in minimal bat or rodent pathology (with many more studies carried out in bats), but this important observation is not mentioned in the text. Finally, the authors lump inoculations into the body in one category which includes subcutaneous, intramuscular, intraperitoneal, intravenous, and other routes

of infection. These are all quite different, as for example, intraperitoneal routes of infection are much more likely to cause pathology than subcutaneous routes of infection, and these should all be considered separately.

We thank the reviewer for acknowledging our additional sensitivity models removing inoculations into the brain. We would like to clarify that all individual-level models include inoculation route as a hierarchical effect rather than fixed effect (detailed in S.1.4.2 and the legend of Figure 2.). As such, the individual estimated effects for each inoculation route are not appropriate to show in our plots of fixed effect estimates (e.g. Figure 2 and all comparable figures for sensitivity models). However, we do separately plot the estimated mean hierarchical effects for our full models (S2.1). Following the Reviewer's suggestion, we have now split inoculations into the body into separate categories of intramuscular, intraperitoneal, and under the skin (subcutaneous, subdermal, or intradermal), resulting in six instead of four inoculation routes (detailed in S1.2.7). We have updated Figure S8 (now Figure S7), which shows the relative severity across inoculation routes and host orders, and includes sample sizes for each combination. This slightly decreased samples sizes for individual models as some studies used a combination of body injections which could not be separated at the individual level. We have re-run all individual-level models and updated all figures. The new results are qualitatively unchanged from our previous models using four inoculation route categories.

4. Analysis of viruses inoculated into both rodents and bats: In response to my previous critique, the authors attempted to analyze a subset of the data containing viruses inoculated into both rodents and bats. However, the analysis undertaken, which used the same approach to analyze these outcomes collectively, is not sufficient. What I had in mind was a kind of paired design, where the resulting pathology from a given virus in both bats and rodents was evaluated at a variety of doses.

We agree with the reviewer that this 'paired design' experiment would be a very powerful test, however, such experiments are rarely if ever carried out in the field. The suggestion, while interesting, is therefore not realistically achievable. We have carried out the closest possible analysis with the currently available data and this provides no reason to doubt the conclusions of our analysis of the full dataset. Beyond the additional analyses added at the previous revision, the best we can do for this suggestion is to expand the discussion on the need for future studies to conduct 'paired' experiments, along with the challenges of those experiments (Lines 336-342):

"Future comparisons of disease outcomes across host groups should inoculate representative species from each group with the same sets of viruses, comprising those that are both homologous and heterologous with a paired design involving the same doses and inoculation routes. Although it would incur major logistical challenges and

costs, such experiments would ideally be carried out using non-invasive proxies for viral load (e.g. using bioluminescent viruses (Stading et al. 2016)) and would observe animals over sufficient timescales to quantify multiple impacts of infection on host fitness."

5. Comparison limited to rodent hosts for viral infections: While I recognize that every meta-analysis has its limits, I find it less conclusive to present comparisons limited to those between bats and rodents only, given that both are notable viral reservoir hosts. The authors suggest that these present clear contrasts in life history evolution, but this is not always the case, as some rodents (e.g. NMRs) have much more "bat-like" phenotypes with respect to longevity and might also be expected to demonstrate considerable viral tolerance. In addition, the assumption that long lifespan should select for tolerance is not always accurate and has been demonstrated in several theoretical pieces (e.g. Boots et al. 2013 Parasite Immunology; Miller et al. 2007 Evolution).

We have addressed this point our manuscript when stating that studies in other taxa would be valuable, and that the life history differences among bats and rodents are not absolute. The two papers suggested by the reviewer are now cited in the introduction when acknowledging the complexity of life history evolution and viral suppression mechanisms (Lines 71-75):

"However, on average rodents have shorter life expectancies and higher reproductive rates than bats, which some theory has suggested may make them less likely to evolve mechanisms to suppress to viral disease, though other models have suggested that these relationships may be complex (Albery and Becker, 2021; Hayman, 2019; Han et al., 2015; Luis et al., 2013; Boots et al., 2013; Miller et al., 2007)".

Regarding the use of rodents as a comparison group, this is largely possible because rodents are also reservoirs for large number of zoonoses, creating an adequate sample size of experiments. The practical implication of limiting our study to two groups is that both bats and rodents might, in principle, both have exceptionally viral co-existence relative to other groups. In the revised Discussion, we explain this possibility and why we think it is unlikely to explain our findings (Lines 294-304):

"While these mammalian groups are commonly used in experimental viral infections and -- as the two largest mammalian orders -- host the majority of zoonotic viruses (Mollentze and Streicker, 2020), the omission of other host clades opens the possibility that both bats and rodents experience unusually mild disease. However, given that the life history strategy of rodents prioritizes reproduction over longevity (Albery and Becker, 2021; Hayman, 2019; Han et al., 2015; Luis et al., 2013), and mechanisms that limit the severity of viral infections in bats are suggested to be byproducts of adaptations to powered flight (Baker et al., 2013; Brook and Dobson, 2015; Calisher et al., 2006; O'Shea et al., 2014; Shen et al., 2010; Wang et al., 2011; Wong et al., 2007), this outcome seems unlikely. Although our results should be re-visited as inoculations into other host

orders accumulate, we suggest that bats and rodents are well characterized groups that represent broader phenomena of taxon-specific immunological traits (Shaw et al., 2017) and context-dependent outcomes of host-virus interactions."

More minor, line-by line comments (with some overlap with above) are as follows:

Line 29-30: ASC2 also reduced inflammatory cytokines caused by orthoreovirus and flaviviruses infections as well.

Added

L54: please clarify that resistance necessitates an improvement in host health from inhibiting infection

We respectfully disagree that while resistance should inhibit or reduce pathogen burden, it does not always necessitate an improvement in host health as resistance mechanisms can sometimes trigger harmful inflammation or tissue damage that reduces host health despite a shrinking infection load.

L63-65: As noted above in #1, this is an inaccurate definition of tolerance. While I agree that many papers are not rigorous and specific in the application of this term, this does not make it ok to continue to perpetuate its use in this way or to draw erroneous conclusions about tolerance using an imprecise definition.

As discussed above we have re-framed out manuscript around disease outcomes rather than tolerance.

L150-153: The authors report the fascinating results that "infection by phylogenetic generalists (i.e., viruses that infect evolutionarily diverse host species) was less likely to result in disease, mortality, and severe disease" and reference Fig. 2. This is a fascinating and important finding, but I was at first confused without seeing clear indication of this predictor variable in Figure 2. Upon re-review of the text, I think that the predictor term 'host phylogenetic diversity' captures this virus generalism but it is not intuitive: it would help to have some part of this term including the word "virus" so that we understanding this is a predictor variable focused on the virus and not the host

In the literature on host specificity, it is common to use refer to phylogenetic distances among hosts, or host phylogenetic diversity. To keep consistent with this literature, we feel that this is the correct term to use in this instance, and provide a detailed description of how this metrics is calculated in the Methods. The caption for figure 2 also explains that 'host phylogenetic diversity' is a viral trait.

L199-201: As noted above in #2, lack of host order effect in dose-pathology interaction could result if the experiments considered all commenced with doses greater that those relevant to natural infections.

As discussed above in response to comment #2, our manuscript presents an analysis of currently available data on experimental inoculations, however we suggest that future studies investigating natural immune responses aim to use realistic doses and inoculation routes, when known.

L201-203: As noted above in #3, the authors refit the models after removing inoculations to the brain, but did the authors fit the models independently for each route of inoculation, comparing pathology subcutaneous infections (at a specific dose) only to other subcutaneous infections and so on? As is, this appears to be comparing apples to oranges.

As discussed above, we have expanded the number of inoculation routes, but only fit sensitivity analyses removing inoculations into the brain as these are known to have severe outcomes and are disproportionately conducted across bat and rodent hosts (See updated Figure S8 (now Figure S7) which shows the relative severity across inoculation routes and host orders, and includes sample sizes for each combination).

L224-227: The authors acknowledge that their analysis might not detect cases where bats experience less-than-expected disease for bat associated viruses. Since it appears that bat experiments are more likely to use homologous viruses and that bat-associated viruses cause higher human CFRs than rodent, it seems like missing this unique bat tolerance might severely underrepresent an important element of their virus tolerance.

As discussed in this section of the manuscript, the raw data suggests that experiments inoculating bats more often used viruses with higher CFRs compared to experiments inoculating rodents, but this effect was not robust after controlling for virus-level hierarchical effects. We also failed to find a difference in the CFRs of viruses used in bats versus rodents when restricting the analysis to heterologous inoculations or when excluding bat-associated viruses. As such, if a genuine reduction of disease in bats was undetectable in our dataset due to biases in viruses selected for experiments, it would primarily reflect bats experiencing less-than-expected disease from bat-associated viruses -- consistent with the expectation that evolutionary association leads to virus-specific adaptations.

In our Implications for Future Research we further explain (Lines 333-336): ***"we identify major evidence gaps in the current body of experimental inoculations: few bat-associated viruses have been inoculated into rodents, rodent-associated viruses with moderate human CFR have not been inoculated into bats or wild rodents, and relatively***

few human-associated viruses have been inoculated into either bats or rodents (Fig. S34)"

L240: still comes back to virus tolerance

As discussed above we have re-framed our manuscript around disease outcomes rather than tolerance.

Line 257-259: Again, Ahn et al. 2023 found that ASC2 functions at the organismal level when added to mice and reduces inflammation and mortality.

As discussed above, our results do not imply that new antivirals will not be discovered from studies of bat immune systems, but that bats are unlikely to be exceptionally fruitful sources of antivirals compared to other host groups. We have given ACS2 as a clear example of a success of this approach.

L262-264: this is all irrelevant considering infectious doses in experimental infections that are well above natural mortality rate
"Passaging virus should, if anything, diminish pathology in bat or rodent host" – isn't this only true for homologous infections?

As discussed above and in the manuscript, considering that infectious doses and mortality rates in natural infections are not well documented and difficult to gather for wild bats, our study analyses all available data on experimental inoculations and adjusts for variation in experimental design, which we believe to be the most appropriate way to rigorously test the paradigm that bats have unusual ability to suppress disease outcomes resulting from viral infections.

In this section, we do note that the outcomes of virulence evolution following serial passage depend on many factors. While the quoted line above does not appear in the manuscript verbatim, our previous and latest manuscript versions present theory suggesting that passaging in alternate hosts decreases severity of disease in original hosts (Lines 285-293):

"Although the degree and mechanisms of pathogen evolution following serial passage depend on many factors (Tsugawa et al., 2014; Elena et al., 2025; Pe˜na et al., 2016), serial passage often increases virulence in passaged hosts and attenuates virulence in original hosts (Kubinak et al., 2015; Lezcano et al., 2023; Ebert, 1998). The viruses in our study were most often passaged in either primate or rodent sources, rather than bats (Fig. S11), which might be expected to decrease, not increase the severity of disease outcomes in inoculated bats. Where available, the mean number of prior passages also did not differ for bat and rodent inoculations (Fig. S11), suggesting that our inability to recover effects of host order on disease outcomes is unlikely attributable to systematic

biases in passaging practices. Nevertheless, future studies should examine how passage number, host origin, and passaging mechanisms affect disease outcomes."

Figures:
Figure 1: should be corrected by dose and somehow differentiated by route of inoculation (maybe colored borders on the boxes?)

Figures 1 and S13 show maximum and mean disease severity per host-virus interaction including both species-level and individual-level data. They are designed to show the taxonomic scope of the raw disease outcome data available rather than a model-corrected prediction. Results that adjust for dose are explored through multiple hierarchical regressions (Section S2.2.5). We therefore feel that modifying Figure 1 to adjust for dose would not adequately represent the raw data.

Figure 2: why is there not a predictor by inoculation route for the individual models? Is that what is shown in Fig. S18? If so, then it needs to be made clear in Fig. 2 that missing components of the model are summarized in Fig S18, and it would be great to know which inoculation route is characterized by which dot. Additionally, the species-level models in Fig. 2 should be subdivided in the analysis by inoculation route and evaluated only against others with the same inoculation route.

As discussed above, all individual-level models include inoculation route as a hierarchical effect rather than fixed effect (detailed in S.1.4.2 and the legend of Figure 2.). As such, the individual estimated effects for each inoculation route are not appropriate to show in our plots of fixed effect estimates (e.g. Figure 2 and all comparable figures for sensitivity models). However, we do separately plot the estimated mean hierarchical effects for our full models (S2.1).

Figure 3: also needs to be scaled by dose and inoculation route

As with Figure 1, Figure 3 shows the scope of the raw data available across host-virus combinations including studies for which individual-level data are not available. As dose is included per individual rather than per host-virus combination, modifying Figure 3 to adjust for dose would not adequately represent the taxonomic scope of the raw data. Separating the figure by inoculation route would overly complicate the figure and might imply that we are presenting a model outcome. We believe it is most useful to show the raw data here.

Notes on supplemental materials:

- As noted above, injection into the body other than the brain lumps subcutaneous, intramuscular, intraperitoneal, intravenous, and other routes of infection. These are quite

different, as for example, intraperitoneal routes of infection are much more likely to cause pathology than subcutaneous routes of infection

As discussed above, we have fitted new models which expanded the inoculation routes as per the reviewers suggestion.

- The authors note that "dose quantities were not reported for natural infections and treated and could not be included in individual-level models adjusting for relative dose"… These seem like the most relevant studies to examine so their absence from the main analyses is troubling… especially since the majority were bat infections with low pathology. This restriction should be noted in the main text.

Four of the 137 studies reporting some aspect of disease outcome, only 4 included experimental inoculation via "natural" routes, with low severity observed in both bats and rodents. Considering the low sample size and lack of difference among host groups, if dose information were available it is unlikely that including these studies would change the main conclusions of our manuscript. We have however included an updated Figure S7 which includes sample size and severity information across inoculation routes for both bats and rodents.

- Fig S8: As noted above in multiple places, I would love to see the analysis where pathology for taxa are compared within route of inoculation: it looks like oronasal routes of infection (which seem the closest to natural possible) are producing less severe pathology in bats than rodents but of course I can't evaluate statistically from the plot and certainly can't account for dose in these cases. Similarly, natural infection routes appear to result in minimal bat or rodent pathology, but this is not otherwise acknowledged in the paper. Finally, as stated above, it is hard to understand what is going on for the body injections since there are such a diversity of different routes lumped together.

We have updated Figure S8 (now S7) to show this information.

- Fig S9 is useful: it would be nice to mention in the discussion that not all clades of bats and rodents are represented. Notably, the Rhinolophid bats (reservoirs for sarbecoviruses) appear to be missing from all of the bat experimental infections. Particularly as only certain clades of bat have been shown to be associated with high CFR zoonotic viruses (Cummings et al. Communications Biology 2025), it seems possible that specific clades of bat or rodent might be uniquely viral tolerant.

We now explicitly mention these clade-level sampling gaps in the main manuscript (Lines 106-109):

"Nevertheless, while some host clades were not subjected to experimental inoculations, our dataset comprised a phylogenetically representative sample of both bats and rodents

(Fig. S8), capturing variation in host life history traits hypothesized to influence disease outcomes (Hayman 2019; Fig. S10)."

- Fig S14 seems more relevant than Fig 1… Why report the max instead of the mean? It is somewhat useful to know if there is the possibility for an infection to become so severe, but the average severity tells us more about the population level tolerance, whereas max severity could be due to poor individual condition in a way that isn't representative of the species.

As discussed in our previous response letter, in response to a reviewer suggestion we provide an alternative version of this plot showing mean severity per host-virus interaction (now Fig S13), but find that the overall pattern of variation in disease severity across bat-virus interactions remains. If the editor prefers that we replace Fig 1 with Fig S13 in the main text we would be happy do so since their conclusion is the same.

- The authors state: "To include doses measured through different dose units (e.g., Mouse LD50, TCID50, PFU), dose/g was log-transformed, then standardized and centered relative to the mean and standard deviation per dose unit." Are you sure this is adequate? It seems that PFUs were more frequently used in rodent vs. bat infections and TCID50 and FFUs in bat vs. rodent. I think your approach is saying that you scaled the mean of each dose unit to be equal, but is there any evidence to suggest that the mean PFU dose is comparable to the mean TCID50 dose, etc?

Exact relationships between dose units were unavailable for most viruses, making it impossible to harmonize dosages into a single comparable scale. Instead, we standardized to units of standard deviation within each dose category and include a hierarchical effect of dose category in models adjusting for relative dose. The inclusion of the hierarchical effect is included to adjust for any potential differences across units, as the reviewer suggests. This is detailed in S1.2.7 and S1.4.2.

- Fig S18 shows that inoculation route had large and variable impacts on host pathology. As noted above, it would be nice to know which of these dots corresponds to which route of inoculation.

As discussed above, we now include Fig. S7 which shows severity across inoculation routes and host orders, and includes sample sizes as numbers of studies and numbers of individuals for each subset.

- If reservoir match results in increased pathology, as shown in individual models, and bats had almost twice the proportion of reservoir-matched experimental infections, then doesn't

this increase the likelihood that bats would show disease from these infections? Is that what is captured in the host order x reservoir match predictor variable highlighted in Fig S23?

The 80% credible intervals for each of these conditional effects overlap across host groups, indicating that there is no clear evidence that bats are more likely to show disease from reservoir-matched experimental inoculations.

- Fig S21-23 and S26-32: Is there a reason that inoculation route is not included (or maybe just not shown) for the individual models represented here that (1) include the reservoir match and HEI features, (2) exclude lyssaviruses, (3) use uncorrected dose adjustments, (4) exclude brain inoculations, and (5) compare only those viruses inoculated both into rodents and bats

As discussed above, inoculation route is included in all individual-level models as a hierarchical effect apart from the base model (Fig. S19), but estimated hierarchical effects are not included in figures depicting estimated effect sized for continuous predictors.

- Fig S32: As noted above in #4, for the analysis of those viruses inoculated into both hosts, why is there not an added effect of virus ID here? Ideally, we’d want to see some kind of paired analysis where you went, one-by-one, through each virus inoculated into both hosts and showed that, at equivalent doses and routes of inoculation, there was no difference in resulting pathology from infection

Models including only viruses inoculated into both host orders include the same structure of hierarchical effects as full models and other sensitivity models. This includes a taxonomic and non-taxonomic virus-level effects that the reviewer suggests. Please see our earlier response to the suggestion on paired experiments. The goal of our manuscript is to use a comparative approach to identify whether general patterns across bat-virus interactions exists, rather than identifying individual viruses which have some support for increased or decreased disease severity.

- Fig S33: It would be great if these figures could also share how these experimental inoculations compare by dose and inoculation route. Maybe add some faceting by inoculation route (or color by inoculation route and facet by host) and scale point size by inoculation dose

We thank the reviewer for their suggestion. Given the already very large number of supplementary figures and that this idea is not currently discussed among the conclusions or results of the manuscript, we opted to include the raw data and scripts to reproduce or augment these figures should future researchers wish to expand on our analyses.

- Fig S34: again, would love to see how this breaks down by dose and inoculation route

We thank the reviewer for their suggestion and include the raw data and scripts to reproduce or augment these figures should future researchers wish to expand on our analyses.
