# Supplementary material for: Viral disease outcomes are indistinguishable between experimentally infected bats and rodents
Source: PLoS Biol. 2026 Oct 29;24(10):e3004053. doi: 10.1371/journal.pbio.3004053 (PMC13743028; doi:10.1371/journal.pbio.3004053)
Supplement: Peer review history

# Author comment: Author response to Decision Letter 0

**DOI:** 10.1371/journal.pbio.3004053.r001

19 Aug 2026

Attachment Submitted filename: Response_to_reviewers_latest.pdf

# Editor report: Decision Letter 0

**DOI:** 10.1371/journal.pbio.3004053.r002

**Authors:** Melissa Vazquez Hernandez

1 Sep 2026

Dear Max, dear Daniel,

Thank you for submitting your manuscript entitled "Viral disease outcomes are indistinguishable between experimentally infected bats and rodents" for consideration as a Research Article by PLOS Biology under our Portable Peer Review scheme. Apologies for the delay.

Your manuscript and previous peer review has now been evaluated by the PLOS Biology editorial staff as well as by an academic editor with relevant expertise and I am writing to let you know that we would like to proceed considering the package and without any additional round of review. However, before sending you a minor decision with just some textual requests from the Academic Editor and editorial requests, we need you to complete your submission by providing the metadata that is required for full assessment. To this end, please login to Editorial Manager where you will find the paper in the 'Submissions Needing Revisions' folder on your homepage. Please click 'Revise Submission' from the Action Links and complete all additional questions in the submission questionnaire.

Once your full submission is complete, your paper will undergo a series of checks in preparation for peer review. After your manuscript has passed the checks it will be sent out for review. To provide the metadata for your submission, please Login to Editorial Manager (https://www.editorialmanager.com/pbiology) within two working days, i.e. by Sep 03 2026 11:59PM.

If your manuscript has been previously peer-reviewed at another journal, PLOS Biology is willing to work with those reviews in order to avoid re-starting the process. Submission of the previous reviews is entirely optional and our ability to use them effectively will depend on the willingness of the previous journal to confirm the content of the reports and share the reviewer identities. Please note that we reserve the right to invite additional reviewers if we consider that additional/independent reviewers are needed, although we aim to avoid this as far as possible. In our experience, working with previous reviews does save time.

If you would like us to consider previous reviewer reports, please edit your cover letter to let us know and include the name of the journal where the work was previously considered and the manuscript ID it was given. In addition, please upload a response to the reviews as a 'Prior Peer Review' file type, which should include the reports in full and a point-by-point reply detailing how you have or plan to address the reviewers' concerns.

During the process of completing your manuscript submission, you will be invited to opt-in to posting your pre-review manuscript as a bioRxiv preprint. Visit http://journals.plos.org/plosbiology/s/preprints for full details. If you consent to posting your current manuscript as a preprint, please upload a single Preprint PDF.

Feel free to email us at plosbiology@plos.org if you have any queries relating to your submission.

Best wishes,

Melissa

Melissa Vazquez Hernandez, Ph.D.

Senior Editor

PLOS Biology

mvazquezhernandez@plos.org

# Author comment: Author response to Decision Letter 1

**DOI:** 10.1371/journal.pbio.3004053.r003

1 Sep 2026

Attachment Submitted filename: Response_to_reviewers_latest_auresp_1.pdf

# Editor report: Decision Letter 1

**DOI:** 10.1371/journal.pbio.3004053.r004

**Authors:** Melissa Vazquez Hernandez

9 Sep 2026

Dear Max, dear Daniel,

Thank you for your patience while we considered your revised manuscript "Viral disease outcomes are indistinguishable between experimentally infected bats and rodents" for publication as a Research Article at PLOS Biology as part of our Portable Peer Review format. This revised version of your manuscript has been evaluated by the PLOS Biology editors and the Academic Editor.

Based on our Academic Editor's assessment of your revision, we are likely to accept this manuscript for publication, provided you satisfactorily address the remaining points raised by the Academic Editor (see below). Please also make sure to address the following data and other policy-related requests.

1) Please add to your Competing Interests the following statement “DGS is a member of PLOS Biology’s Editorial Board. The other authors declare that no competing interests exist."

2) Please add the weblink of the funding agencies in the Financial Disclosure statement in the manuscript details.

3) We do not have a word limit. So we would strongly encourage to move the supplementary material and methods, and the supplementary results, to the main text which can provide the readers an easier access to all information.

4) You may be aware of the PLOS Data Policy, which requires that all data be made available without restriction: http://journals.plos.org/plosbiology/s/data-availability. For more information, please also see this editorial: http://dx.doi.org/10.1371/journal.pbio.1001797

Please supply the numerical values either in the a supplementary file or as a permanent DOI’d deposition for all figures

NOTE: the numerical data provided should include all replicates AND the way in which the plotted mean and errors were derived (it should not present only the mean/average values). If the data to reproduce them are in the Zenodo link, please state them in Data Availability

5) Please cite the location of the data clearly in all relevant main and supplementary Figure legends, e.g. “The data underlying this Figure can be found in S1 Data” or “The data underlying this Figure can be found in https://doi.org/10.5281/zenodo.XXXXX”

6) Please provide the tree files for the phylogenetic trees in Figures 1, S8, S13, S36, S37, S38, S39, S40, S41, S42, S43, S44, S45, S46, S47. Please make sure all relevant figures have scale bars.

7) Supplementary files (e.g., excel). Please ensure that all data files are uploaded as 'Supporting Information' and are invariably referred to (in the manuscript, figure legends, and the Description field when uploading your files) using the following format verbatim: S1 Data, S2 Data, etc. Multiple panels of a single or even several figures can be included as multiple sheets in one excel file that is saved using exactly the following convention: S1_Data.xlsx (using an underscore).

8) Please ensure that your Data Statement in the submission system accurately describes where your data can be found and is in final format, as it will be published as written there

As you address these items, please take this last chance to review your reference list to ensure that it is complete and correct. If you have cited papers that have been retracted, please include the rationale for doing so in the manuscript text, or remove these references and replace them with relevant current references. Any changes to the reference list should be mentioned in the cover letter that accompanies your revised manuscript.

In addition to these revisions, you may need to complete some formatting changes, which you will receive in a follow up email. A member of our team will be in touch with a set of requests shortly. If you do not receive a separate email within a few days, please assume that checks have been completed, and no additional changes are required.

We expect to receive your revised manuscript within two weeks.

To submit your revision, please go to https://www.editorialmanager.com/pbiology/ and log in as an Author. Click the link labelled 'Submissions Needing Revision' to find your submission record. Your revised submission must include the following:

- a cover letter that should detail your responses to any editorial requests, if applicable, and whether changes have been made to the reference list

- a Response to Reviewers file that provides a detailed response to the reviewers' comments (if applicable, if not applicable please do not delete your existing 'Response to Reviewers' file.)

- a track-changes file indicating any changes that you have made to the manuscript.

NOTE: If Supporting Information files are included with your article, note that these are not copyedited and will be published as they are submitted. Please ensure that these files are legible and of high quality (at least 300 dpi) in an easily accessible file format. For this reason, please be aware that any references listed in an SI file will not be indexed. For more information, see our Supporting Information guidelines:

https://journals.plos.org/plosbiology/s/supporting-information

*Published Peer Review History*

Please note that you may have the opportunity to make the peer review history publicly available. The record will include editor decision letters (with reviews) and your responses to reviewer comments. If eligible, we will contact you to opt in or out. Please see here for more details:

https://plos.org/published-peer-review-history/

*Press*

Should you, your institution's press office or the journal office choose to press release your paper, please ensure you have opted out of Early Article Posting on the submission form. We ask that you notify us as soon as possible if you or your institution is planning to press release the article.

*Protocols deposition*

To enhance the reproducibility of your results, we recommend that if applicable you deposit your laboratory protocols in protocols.io, where a protocol can be assigned its own identifier (DOI) such that it can be cited independently in the future. Additionally, PLOS ONE offers an option for publishing peer-reviewed Lab Protocol articles, which describe protocols hosted on protocols.io. Read more information on sharing protocols at https://plos.org/protocols?utm_medium=editorial-email&utm_source=authorletters&utm_campaign=protocols

Please do not hesitate to contact me should you have any questions.

Sincerely,

Melissa

Melissa Vazquez Hernandez, Ph.D.

Senior Editor

mvazquezhernandez@plos.org

PLOS Biology

------------------------------------------------------------------------

COMMENTS FROM ACADEMIC EDITOR:

At a couple of key junctures, the phrasing could be less polarizing while still accurate. For example, the authors could use the last sentence of the abstract to emphasize the fascinating basic science findings (e.g., coevolution rules! rather than bats being universally impervious to viruses) instead of focusing on potential translational impacts. I also think that the discussion sentence lines 275-280 is just about the dangers of over-generalization from otherwise well-grounded inferences. But the framing of that sentence around “beliefs” (in both this version and the previous one) rather than well grounded inferences on specific host/virus taxa may have set folks off. Perhaps the authors should consider rephrasing?

# Editor report: Decision Letter 2

**DOI:** 10.1371/journal.pbio.3004053.r005

**Authors:** Melissa Vazquez Hernandez

28 Sep 2026

Dear Max, dear Daniel,

I hope you had a nice weekend.

Thank you for the submission of your revised Research Article "Viral disease outcomes are indistinguishable between experimentally infected bats and rodents" for publication in PLOS Biology. On behalf of my colleagues and the Academic Editor, Andrea L. Graham, I am pleased to say that we can in principle accept your manuscript for publication, provided you address any remaining formatting and reporting issues. These will be detailed in an email you should receive within 2-3 business days from our colleagues in the journal operations team; no action is required from you until then. Please note that we will not be able to formally accept your manuscript and schedule it for publication until you have completed any requested changes.

Please take a minute to log into Editorial Manager at http://www.editorialmanager.com/pbiology/, click the "Update My Information" link at the top of the page, and update your user information to ensure an efficient production process.

PRESS

We frequently collaborate with press offices. If your institution or institutions have a press office, please notify them about your upcoming paper at this point, to enable them to help maximise its impact. If the press office is planning to promote your findings, we would be grateful if they could coordinate with biologypress@plos.org. If you have previously opted in to the early version process, we ask that you notify us immediately of any press plans so that we may opt out on your behalf.

We also ask that you take this opportunity to read our Embargo Policy regarding the discussion, promotion and media coverage of work that is yet to be published by PLOS. As your manuscript is not yet published, it is bound by the conditions of our Embargo Policy. Please be aware that this policy is in place both to ensure that any press coverage of your article is fully substantiated and to provide a direct link between such coverage and the published work. For full details of our Embargo Policy, please visit http://www.plos.org/about/media-inquiries/embargo-policy/.

Thank you again for choosing PLOS Biology for publication and supporting Open Access publishing. We look forward to publishing your study.

Sincerely,

Melissa

Melissa Vazquez Hernandez, Ph.D.

Senior Editor

PLOS Biology

mvazquezhernandez@plos.org
